# *Helicobacter pylori* infection, peptic ulcer, gastritis and risk of Parkinson's disease: A nationwide matched cohort study

**DOI:** 10.1177/1877718X261466875

**Published:** 2026-07-15

**Authors:** Sara Licaj, Alexander Ploner, Agnieszka Szwajda, Sara Hägg, Fang Fang, Jonas F Ludvigsson, Karin Wirdefeldt

**Affiliations:** 1 Department of Medical Epidemiology and Biostatistics, 27106 Karolinska Institutet, Stockholm, Sweden; 2 Institute of Environmental Medicine, 27106 Karolinska Institutet, Stockholm, Sweden; 3 Department of Pediatrics, Örebro University Hospital, Örebro, Sweden; 4 Department of Clinical Neuroscience, Karolinska Institutet, Stockholm, Sweden

**Keywords:** *Helicobacter pylori*, Parkinson's disease, gastritis, peptic ulcer, cohort study

## Abstract

**Background:**

Gastrointestinal symptoms are frequent in Parkinson's disease (PD) and may precede diagnosis by decades, suggesting a possible role in its pathogenesis. Patients with PD have higher rates of *Helicobacter pylori* (*H. pylori*) infection than the general population, although its potential contribution to PD development remains unclear. Similarly, evidence linking gastritis and peptic ulcer to PD risk is limited.

**Methods:**

Using the nationwide ESPRESSO cohort, we investigated associations between *H. pylori* infection, peptic ulcer, gastritis, and future risk of PD. Individuals diagnosed with these conditions between 1987 or 1990 and 2017 were matched to ≤5 referents from the general population by age, sex, calendar year, and county of residence. Stratified Cox models estimated hazard ratios (HRs), adjusting for country of birth, education, chronic obstructive pulmonary disease, and number of healthcare visits. Incident PD was identified from the National Patient Register.

**Results:**

The *H. pylori* cohort included 48,258 exposed individuals and 231,805 referents; the peptic ulcer cohort included 52,761 exposed individuals and 274,313 referents, while the gastritis cohort included 242,325 exposed individuals and 1,053,688 referents. *H. pylori* infection (HR: 1.14, 95% CI: 1.02–1.28) and gastritis (HR: 1.14, 95% CI: 1.09–1.19) were associated with a marginally increased PD risk, but we found no significant association in peptic ulcer (HR: 1.08, 95% CI: 0.98–1.19). We observed increased PD risk up to ten years after gastritis diagnosis and no time-varying association for *H. pylori* and peptic ulcer.

**Conclusions:**

*H. pylori* and gastritis may have some relevance for PD development but are unlikely to be major contributors to its pathogenesis.

**Plain language summary title:**

Using gastrointestinal biopsies to explore the link between *Helicobacter pylori* infection, peptic ulcer, gastritis and Parkinson's disease: findings from a nationwide Swedish cohort study

## Plain language summary

Many patients with PD experience gastrointestinal symptoms long before they are diagnosed with PD. This has led scientists to ask whether gastrointestinal diseases might be linked to the later development of PD. Understanding such a link could help identify people at risk and lead to new strategies for prevention or treatment. In this study, we used information from ESPRESSO (Epidemiology Strengthened by HistoPathology Reports in Sweden), a large Swedish health database which includes detailed records of gastrointestinal biopsies from the entire Sweden plus linkages to several national registers. Persons diagnosed with *Helicobacter pylori* infection, gastritis or peptic ulcer between 1987 or 1990 and 2017 were included in the study. Each person with one of these conditions was compared to persons from the general population without a biopsy, but similar age, sex, and geographic area of residence. Everyone was followed over time to see how many persons in each group were later diagnosed with PD. We found that persons with *H. pylori* infection or gastritis had a slightly higher risk of being diagnosed with PD compared to those without biopsies. The increased risk was greatest within the first two years after a gastritis diagnosis and remained higher for up to ten years. However, we did not find a strong association between peptic ulcer and PD risk. This study suggests a possible link between these gastrointestinal conditions and PD, but since the risk increase was small, they might not be key factors in disease development. We encourage more research to confirm our findings and to determine whether gastrointestinal inflammation or infection reflects early manifestation of PD rather than a causal factor.

## Introduction

Gastrointestinal symptoms are frequent among Parkinson's disease (PD) patients and may start decades before diagnosis.^1^ According to the Braak hypothesis, α-synuclein aggregation can start in enteric neurons and subsequently spread to the central nervous system via the vagal nerve.^2^ The brain-first vs body-first model suggests a similar disease mechanism, as α-synuclein aggregation in the body-first subtype originates in the peripheral nervous system, including enteric neurons, and later propagates to the brain.^3^ Although further validation is needed, the brain-first vs body-first model is supported by a growing body of clinical and experimental evidence.^4–8^ α-Synuclein aggregation has been observed in the vagal nerve and along the gastrointestinal tract in PD patients many years before motor symptom onset.^5,9^ Truncal vagotomy, formerly used to treat peptic ulcer, has been associated with reduced PD risk.^10,11^ A recent cohort study reported a strong association between upper gastrointestinal mucosal damage and risk of PD, further supporting a role of the gut in disease pathogenesis.^12,13^ Gut dysbiosis is also possibly involved in the pathophysiological processes underlying PD, as it increases intestinal permeability, inducing gastrointestinal symptoms.^14,15^

*Helicobacter pylori (H. pylori*) is a gram-negative bacterium identified as a primary cause of peptic ulcer and chronic gastritis, as well as a major risk factor for gastric cancer. It is one of the most prevalent infections worldwide, although most infected individuals remain asymptomatic and without long-term effects.^16,17^ *H. pylori* can potentially be involved in PD pathogenesis, triggering autoimmunity through molecular mimicry and inducing inflammation that may propagate to the brainstem through the gut-brain axis, leading to degeneration of dopaminergic neurons.^1,17,18^ This bacterium might also directly access the brain through the oral–nasal olfactory route, leading to neurotoxicity and neuroinflammation.^17^ Higher infection rates have been reported in patients with PD than in the general population, along with worsening motor symptoms and impaired levodopa absorption following infection.^16,19–25^ One study reported a positive association between *H. pylori* eradication therapy and PD risk, but no associations between gastritis, peptic ulcer, and PD risk.^26^ Another study revealed a positive association between multiple gastrointestinal disorders, including gastritis, and PD, but no significant association with peptic ulcer, despite earlier reports of higher prevalence of ulcers in patients with PD.^27,28^

Although several research findings support a link between *H. pylori* and PD, the available evidence is contradictory.^29–31^ Many studies have focused on the effects or prevalence of *H. pylori* in patients already diagnosed with PD compared with the general population, which may lead to biases. The possible involvement of gastritis or peptic ulcer in PD pathogenesis has been scarcely investigated, and previous studies have reported conflicting results or lacked histopathological data. Therefore, the present research aims to examine the potential role of *H. pylori* infection, gastritis and peptic ulcer in PD risk, by conducting a large-scale population-based study that relies on biopsy-based diagnoses. We hypothesized that *H. pylori* infection, gastritis, and peptic ulcer increase the risk of PD.

## Methods

### Data sources

The study population was derived from the ESPRESSO (Epidemiology Strengthened by HistoPathology Reports in Sweden) cohort, consisting of 6.1 million histopathology records among 2.1 million unique individuals from 28 pathology departments across Sweden between 1965 and 2017.^32^ To obtain additional health and sociodemographic data, the ESPRESSO cohort was linked to Swedish national registers such as the National Patient Register (NPR), Causes of Death Register (CDR), Prescribed Drug Register (PDR) and the Longitudinal Integrated Database for Health Insurance and Labor Market Studies (LISA).^32–34^

### Study design

This study used a population-based matched cohort design, where three distinct cohorts were derived from the independent assessment of each exposure. Exposed individuals were defined as anyone with a biopsy positive for one of the following diagnoses: *H. pylori* infection, peptic ulcer or gastritis. As individuals may have more than one biopsy record, the index date of *H. pylori* infection, peptic ulcer, or gastritis was defined as the first-ever date of biopsy leading to any of these diagnoses. Every exposed individual was matched to up to five referents who were free of gastrointestinal biopsy at the index date of the exposed individual. Referents were randomly selected from the general population and individually matched to the exposed individual on age, sex, calendar year and county of residence. The index date of the exposed individual was used as the index date for their matched referents. Owing to the dynamic nature of the cohort, unexposed individuals could be matched multiple times. Those who transitioned to exposed status were excluded from further matching and re-indexing. Additional details on exposure trajectories in our study cohort and re-indexing can be found in Table S1.

To ensure the capture of relevant information on outcome and covariates, only individuals who had lived continuously in Sweden for at least 5 years before the diagnosis date of the respective gastrointestinal disease were included in the study. Individuals with an NPR-based PD diagnosis before the exposure diagnosis date were excluded, as we were only interested in the incident diagnosis of PD. We further excluded individuals who died before the index date and those aged 35 years or younger at the index date, as PD diagnosed at a very young age is typically related to genetic causes.

As the NPR reached nationwide coverage for inpatient care in 1987, the start of the study period for gastritis and peptic ulcer was 1987-01-01, to ensure reliable PD detection.^35^ For *H. pylori* infection, we set the study start in 1990-01-01, as diagnoses before this time were uncommon in Sweden and were judged to be unreliable. In all three cohorts, follow-up started at the index date until date of PD diagnosis, 31 December 2021, death, emigration or re-indexing date, whichever came first.

### Exposure ascertainment

*H. pylori* infection was identified in ESPRESSO by topography codes ‘T63’ (stomach) and ‘T64’ (duodenum) and biopsy SNOMED codes starting with ‘ME137’ or ‘E137’; peptic ulcer was identified by topography codes ‘T63’ and ‘T64’ and biopsy codes starting with ‘ME38’; gastritis was identified by topography code ‘T63’ and biopsy codes starting with ‘M40’, ‘M41’, ‘M42’, ‘M43’, ‘M44’, or ‘M463’ or ‘M47’. Among individuals with *H. pylori* infection, gastritis, or peptic ulcer identified by biopsy in ESPRESSO, separately for each condition, we calculated the proportion of corresponding diagnoses in the NPR.

### Outcome ascertainment

Incident PD was defined by diagnoses in the NPR according to the following Swedish International Classification of Diseases (ICD) codes: 342.00 (ICD-8: 1968–1986), 332.0 (ICD-9: 1987–1996), and G20 (ICD-10: 1997 onwards). Both inpatient and outpatient contacts were included, as well as primary and secondary diagnoses in the NPR. For individuals with multiple PD diagnoses, the earliest recorded diagnosis date was considered the diagnosis date of PD. The accuracy of PD diagnoses in the NPR has previously been reported to be 70.8%, increasing to 83.0% when diagnoses are restricted to primary diagnoses only.^36^

### Covariates

In addition to the matching variables, we included additional variables that could confound the associations between the gastrointestinal conditions under investigation and the risk of PD. The country of birth was included and coded into ‘Sweden’ and ‘Other’. Information on the highest educational attainment at the index date was retrieved from LISA and educational levels were recoded into three levels: compulsory education (≤ 9 years), upper secondary education (10–12 years), and college or university (≥ 13 years) or missing for individuals with no education information. To control for surveillance bias, the number of specialist care contacts (both inpatient and outpatient) was also included from the NPR. We considered visits within 5 years and up to 6 months before the index date, dividing them into the following categories: 0, 1, 2, 3–5 and ≥ 6.

As smoking is negatively associated with PD, but positively associated with *H. pylori *infection, gastritis and peptic ulcer, we included chronic obstructive pulmonary disease (COPD) diagnoses as a proxy for heavy smoking.^37–40^ We retrieved them from the NPR via ICD codes 491, 492, 496 (ICD-9) and J41–44 (ICD-10). Only the first COPD diagnoses after age 40 were included, as disease onset before this age is likely caused by factors other than smoking. Both prevalent COPD diagnoses at baseline and diagnoses occurring during follow-up were considered; in the latter case, we treated COPD as a time-varying exposure and split the time at risk at the first COPD diagnosis after baseline.

### Statistical analysis

We reported descriptive statistics for both exposed and unexposed individuals for all variables at the start of the study period. The median years of follow-up, crude PD incidence rates and crude mortality rates were also included.

We quantified the associations between *H. pylori* infection, peptic ulcer, gastritis and the risk of PD as hazard ratios (HRs) with 95% confidence intervals (CIs) by comparing the exposed individuals to the unexposed ones. Estimates were based on Cox regression models with attained age as the underlying time scale, stratified by matched index sets. All models were adjusted for country of birth, educational level, number of healthcare contacts prior to the index date, and COPD.

We assessed the proportional hazard assumption for all three cohorts via Schoenfeld residual tests and plots. Based on this evidence, we modelled the associations of *H. pylori* infection and peptic ulcer with PD as time-constant associations, whereas the association between gastritis and PD was modelled as a time-varying and piecewise constant association, with separate HRs (95% CIs) estimated for 0-≤2 years, 2-≤5 years, 5-≤10 years, 10-≤15 years, 15-≤20 years, and more than 20 years after the index date.

In a sensitivity analysis, all models were refitted using only inpatient or outpatient hospital contacts with PD as the primary diagnosis as the outcome.

P-values below 0.05 from a two-sided test were interpreted as statistically significant. All statistical analyses were performed via R (4.3.1).^41^

## Results

**Figure 1.**
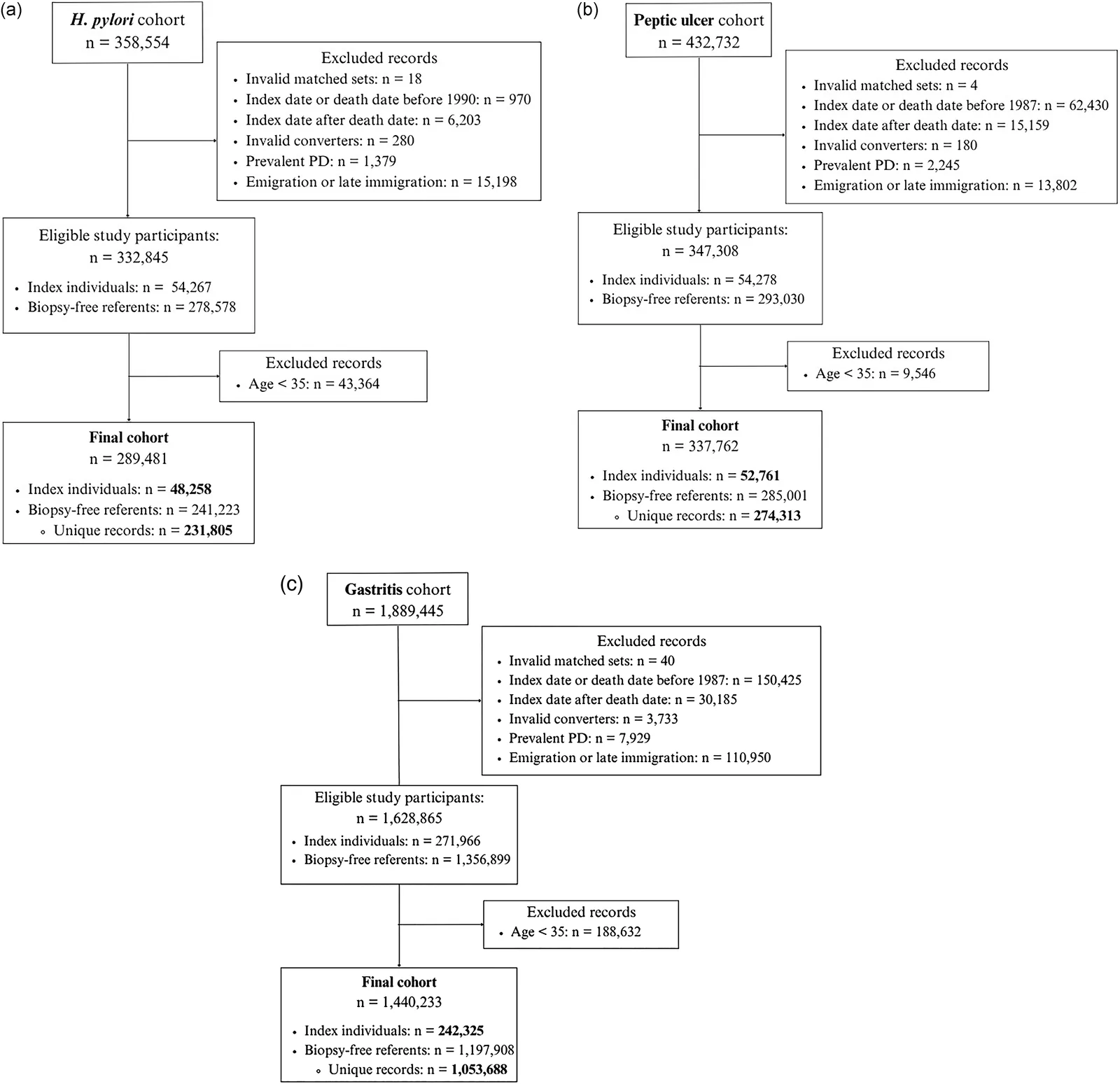
Triple flowchart of the three ESPRESSO cohorts under analysis. The invalid converter patterns are presented in Table S1. **a** Flowchart for the *H. pylori* cohort. **b** Flowchart for the peptic ulcer cohort. **c** Flowchart for the gastritis cohort.

The initial cohort sizes and data exclusion flows are presented in Figure 1.

Table 1 summarizes the three cohorts included in the study. The *H. pylori* cohort was the smallest, including 48,258 exposed individuals and 231,805 matched referents. The peptic ulcer cohort included 52,761 exposed individuals and 274,313 referents, whereas the gastritis cohort consisted of 242,325 exposed individuals and 1,053,688 referents. There was an overlap among exposed individuals across the cohorts. Specifically, 3397 study participants were diagnosed with all three conditions. Among individuals diagnosed with *H. pylori* infection, 42,990 (89%) were also diagnosed with gastritis, while 4417 (9.2%) were also diagnosed with peptic ulcer. There were 23,971 study participants diagnosed with both peptic ulcer and gastritis, corresponding to 45.4% of individuals diagnosed with peptic ulcer and 9.9% of those diagnosed with gastritis. Among those with *H. pylori* infection*,* 82% received their diagnosis on the same date as they received a diagnosis of either gastritis or peptic ulcer, or both, suggesting that these conditions were detected during the same endoscopic examination.

**Table 1.** Matched study cohorts in ESPRESSO listing characteristics of index individuals and their matched biopsy-free referents.

	Cohort 1		Cohort 2		Cohort 3	
	*H. pylori*	Biopsy-free^a^	Peptic ulcer	Biopsy-free^a^	Gastritis	Biopsy-free^a^
Total, n (%)	48,258 (100)	231,805 (100)	52,761 (100)	274,313 (100)	242,325 (100)	1,053,688 (100)
Female, n (%)	25,952 (53.8)	124,393 (53.7)	27,267 (51.7)	140,587 (51.3)	128,280 (52.9)	557,932 (53)
Born in Sweden, n (%)	30,022 (62.2)	195,326 (84.3)	46,669 (88.5)	248,730 (90.7)	194,776 (80.4)	930,747.0 (88.3)
Age at index date, mean (SD)	63.3 (14.1)	62.3 (14.2)	69.0 (13.0)	69.0 (13.0)	65.3 (13.7)	63.9 (13.7)
Years of follow-up, median (IQR)	7.9 (3.9–13.2)	8.0 (4.1–13.3)	6.7 (2.4–13.4)	9.3 (4.4–16.1)	8.8 (3.8–15.1)	8.7 (4.1–14.7)
Educational attainment, n (%)						
≤ 9 years	18,393 (38.1)	73,188 (31.6)	26,041 (49.4)	121,640 (44.3)	103,230 (42.6)	408,038 (38.7)
10–12 years	18,256 (37.8)	89,841 (38.8)	15,869 (30.1)	82,288 (30.0)	83,634 (34.5)	367,779 (34.9)
≥ 13 years	10,461 (21.7)	64,289 (27.7)	5310 (10.1)	41,896 (15.3)	41,177 (17.0)	220,675 (20.9)
Missing	1148 (2.4)	4487 (1.9)	5541 (10.5)	28,489 (10.4)	14,284 (5.9)	57,196 (5.4)
COPD^b^, n (%)	5187 (10.7)	17,275 (7.5)	8041 (15.2)	24,075 (8.8)	29,838 (12.3)	84,751 (8.0)
N. healthcare contacts^c^, n (%)						
0	13,196 (27.3)	93,576 (40.4)	17,681 (33.5)	131,217 (47.8)	77,241 (31.9)	509,344 (48.3)
1	6995 (14.5)	35,051 (15.1)	8520 (16.1)	45,029 (16.4)	38,588 (15.9)	169,636 (15.9)
2	4767 (9.9)	21,679 (9.4)	5350 (10.1)	25,509 (9.3)	24,446 (10.1)	94,532 (9.0)
3–5	8672 (18.0)	35,673 (15.4)	8739 (16.6)	36,366 (13.3)	40,515 (16.7)	137,335 (13.0)
≥ 6	14,628 (30.3)	45,826 (19.8)	12,471 (23.6)	36,192 (13.2)	61,535 (25.4)	142,841 (13.6)
Incident PD, n (%)	412 (0.9)	1871 (0.8)	579 (1.1)	3635 (1.3)	2731 (1.1)	11,638 (1.0)
Incidence rate, per 1000 py	1.0	0.9	1.3	1.2	1.1	1.0
Mortality rate, per 100 py	4.9	4.2	9.1	6.2	5.9	5.2

^a^ Individuals without a biopsy-based diagnosis of *H. pylori*, peptic ulcer and gastritis respectively, matched from the Swedish general population on age at index date, sex, calendar year of biopsy and county of residence.

^b^ Number of chronic obstructive pulmonary disease (COPD) diagnoses at baseline or during follow-up.

^c^ Number of inpatient and outpatient healthcare contacts, within 5 years and up to 6 months before the index date.

SD: standard deviation; IQR: interquartile range; COPD: chronic obstructive pulmonary disease; PD: Parkinson's disease; py: person years.

When comparing diagnoses of *H. pylori* infection, gastritis or peptic ulcer in ESPRESSO with those in the NPR, we found that most individuals with a diagnosis in ESPRESSO did not have a corresponding diagnosis in the NPR (Table S3). Among unexposed individuals in ESPRESSO, above 94% were also classified as unexposed in the NPR.

Overall, the three cohorts had similar proportions of females. The number of individuals born abroad was considerably higher among individuals with *H. pylori* infection (37.8%) than among their matched referents (15.7%). Similarly, but less markedly, differences were noted in the peptic ulcer and gastritis cohorts. Due to matching, the age and sex distributions were similar between exposed individuals and their matched referents within each cohort. However, individuals in the peptic ulcer cohort were on average older at the index date (69 years) than those in the *H. pylori* (62–63 years) and gastritis (64–65 years) cohorts. The median follow-up time ranged between 6.7 and 9.3 years across the three cohorts.

The distribution of educational attainment showed slight differences among the cohorts. The individuals in the peptic ulcer cohort had the highest proportion of low-educated participants (49.4% for individuals with peptic ulcer, 44.3% for referents), whereas the *H. pylori* cohort had the lowest proportion of low-educated individuals (38.1% for biopsy-positive individuals and 31.6% for referents). COPD before or after the index date was most common among exposed individuals; the disease was diagnosed in 15.2% of participants with peptic ulcer (8.8% for referents), 12.3% of individuals with gastritis (8.0% for referents) and 10.7% of *H. pylori-*infected individuals (7.5% for referents). The number of healthcare contacts during the 5 years before the index date varied significantly across the three cohorts. Exposed individuals generally had more healthcare contacts than their matched referents, and the largest number of healthcare contacts was found among individuals with *H. pylori* infection.

The number of incident PD diagnoses was 2283 in the *H. pylori* cohort, 4214 in the peptic ulcer cohort, and 14,369 in the gastritis cohort. The incident PD rates per 1000 person-years were 1.0 for the *H. pylori*-exposed cohort (0.9 for referents), 1.3 in the peptic ulcer exposed cohort (1.2 for referents) and 1.1 for individuals exposed to gastritis (1.0 for referents). Mortality rates, however, were higher in the exposed groups than in the reference group across all cohorts, with peptic ulcer showing the highest rate (9.1 per 100 person-years).

For *H. pylori* and peptic ulcer, Schoenfeld residuals plots, and correlation tests did not provide evidence of non-proportionality, meaning that the effect of these exposures on PD hazard can be modelled as constant over time. The main analysis revealed a marginal association between *H. pylori* infection (HR: 1.14, 95%CI: 1.02–1.28) and an increased risk of developing PD later in life, with no statistically significant association for peptic ulcer (HR: 1.08, 95%CI: 0.98–1.19) (Table 2). For simplicity of presentation, the time-constant HR for gastritis was 1.14 (95%CI: 1.09–1.19).

**Table 2.** Results of the multivariate Cox regression analysis stratified by matched sets, with attained age as primary time scale, adjusted for country of birth, education, chronic obstructive pulmonary disease (COPD) and number of healthcare contacts.

Model	Exposure status	HR^a^	95% CI^a^	p-value
*Time constant*	*H. pylori-*	—	—	
	*H. pylori+*	1.14	1.02, 1.28	0.026
	Peptic ulcer-	—	—	
	Peptic ulcer+	1.08	0.98, 1.19	0.12
	Gastritis-	—	—	
	Gastritis+	1.14	1.09, 1.19	<0.001
*Time-varying*	**Time since gastritis diagnosis**			
	Gastritis-	—	—	
	0 - ≤ 2 years	1.28	1.16, 1.41	<0.001
	2 - ≤ 5 years	1.16	1.06, 1.27	0.002
	5 - ≤ 10 years	1.14	1.05, 1.25	0.003
	10 - ≤ 15 years	1.07	0.95, 1.20	0.3
	15 - ≤ 20 years	0.94	0.80, 1.10	0.4
	20+ years	1.01	0.80, 1.28	>0.9

^a^ HR: Hazard Ratio, CI: Confidence Interval

For gastritis, we observed time-varying effects, with the highest increase in PD risk occurring within the first two years after a gastritis diagnosis (HR: 1.28; 95%CI: 1.16–1.41). This association remained statistically significant for up to ten years after the diagnosis (Table 2).

Higher educational levels and being born outside Sweden were associated with increased PD risk, particularly in the gastritis cohort (Table S2). An inverse association was also suggested between COPD and PD in all three cohorts. In terms of healthcare contacts, an increased risk of PD was observed in relation to a higher number of contacts in the gastritis and peptic ulcer cohorts, but not in the *H. pylori* cohort.

Restricting to the definition of PD as hospital contact with PD as the primary diagnosis only, the results were unchanged in the gastritis cohort, but the HR estimates decreased in the *H. pylori* infection and peptic ulcer cohorts (Table S4).

## Discussion

This population-based matched cohort study showed marginally increased PD risk estimates for *H. pylori* infection and gastritis, but no statistically significant association with peptic ulcer. Exposure to gastritis was associated with an increase in PD risk up to ten years after diagnosis, while there were no time-varying effects for *H. pylori* infection and peptic ulcer.

There are several potential explanations for our results. Gastritis is characterized by inflammation of the gastric mucosa, with a range of potential causes including infections, autoimmune responses, and the use of nonsteroidal anti-inflammatory drugs.^42^ Previous studies have identified α-synuclein aggregates in the stomach wall of both confirmed and presymptomatic patients with PD, suggesting that the upper gastrointestinal tract may be involved in PD pathogenesis.^2,4^ In the present study, we observed an increased risk of PD within the first two years following a gastritis diagnosis and up to ten years thereafter. These findings may suggest that acute inflammation plays a more prominent role in triggering disease-related mechanisms. However, the lack of an association with peptic ulcer does not support this hypothesis. While gut inflammation is recognized as a factor contributing to PD development, the underlying inflammatory mechanisms involved and their effects on gut-brain communication are not fully understood.^43,44^ Gastrointestinal inflammation is closely related to gut dysbiosis and increased intestinal permeability, with the enteric nervous system serving as a key mediator.^45^ Disruption of the intestinal barrier allows the translocation of bacteria and endotoxins that can induce an immune response, which is marked by the release of proinflammatory cytokines.^45^ *H. pylori* infection can trigger this cascade by damaging the gastric epithelium and inducing mucosal inflammation.^46^ The subsequent immune response of enteric neurons may compromise the integrity of the blood-brain barrier and activate glial cells, driving neuroinflammatory and neurodegenerative processes involved in PD pathogenesis.^47,48^ In addition, *H. pylori* infection can contribute to disease development by altering the composition of the gut microbiota.^49–51^ An alternative explanation for our results is that *H. pylori* mediates pathophysiological changes in PD progression, rather than being a risk factor itself. Several studies indicate that the infection is associated with worse motor fluctuations and impairments in levodopa absorption in patients with PD.^52^ Reverse causation might also explain our results, as gastrointestinal symptoms are frequent in patients with PD and there is often a delay between the clinical PD onset and diagnosis recorded in the register.^36^ Our findings might also reflect early prodromal symptoms of PD, such as impaired gut physiology or immune response, leading to increased susceptibility to gastrointestinal pathology.^4^ Prodromal gastrointestinal symptoms can contribute to increased healthcare seeking, potentially leading to an earlier diagnosis of PD. Although we tried to control for surveillance bias by adjusting for number of healthcare visits, we cannot exclude the possibility that such bias influenced our findings.

Previous studies examining the associations between *H. pylori*, peptic ulcer, gastritis, and PD have yielded conflicting results. A meta-analysis involving 33,125 participants reported a moderately increased PD risk among *H. pylori-*positive individuals (pooled OR: 1.59, 95% CI: 1.37–1.85).^53^ Compared with these results, our study showed a less pronounced but still marginally increased PD risk in relation to *H. pylori* infection. However, the meta-analysis included no cohort studies and only one study that used histologic examination.^53,54^ Discrepancies between our results and the meta-analysis might be explained by our use of biopsy data to ascertain exposures, differences in study design, geographic factors and healthcare systems. Moreover, the overall findings of the meta-analysis were largely based on a Danish register-based case-control study, which found increased PD risk following *H. pylori* eradication treatment prescribed at least five years before diagnosis.^26^ Interestingly, the authors did not observe similar associations for gastritis or peptic ulcer diagnoses, but reported a positive link between proton pump inhibitor use and later PD.^26^ Compared to our results, these discrepancies suggest that the exposure ascertainment and covariates included in the statistical analysis can significantly influence risk estimates. A Taiwanese population-based cohort study found a strong positive association between *H. pylori* and PD.^54^ Despite similarities in exposure assessment, their results were based on a significantly restricted number of PD cases, which might have influenced the magnitude of the observed risk estimates; the authors did not find an effect of eradication therapy for *H. pylori* infection, which contrasts with the Danish findings.^54^ Our results aligned with a case-control study reporting a positive association between gastritis and subsequent PD, but not with peptic ulcer.^27^ A retrospective cohort study found a strong association between upper gastrointestinal mucosal damage – including ulcer – and PD risk, but not among those with a history of *H. pylori* infection on endoscopy.^12^ Similarly, a recent Mendelian randomization study found no causal association between *H. pylori* infection and PD, but reported an association between infection-related antibodies and clinical presentation of PD.^29^ Thus, gastrointestinal pathology rather than the presence of *H. pylori* may contribute to neurodegenerative processes through mechanisms such as increased intestinal permeability and subsequent systemic inflammation.^45^

This study has several strengths. The large-scale and high-quality Swedish registers allow for comprehensive population coverage that minimizes selection bias. The prospective cohort design and long follow-up period enhance the validity and generalizability of our findings. In addition, the biopsy-confirmed exposure assessment strengthens the accuracy of exposure classification, minimizing the risk of false positives. Only a small proportion of exposed individuals in ESPRESSO was classified as exposed also in the NPR, suggesting that ESPRESSO is a better data source to capture *H. pylori* infection, gastritis and peptic ulcer diagnoses compared to the NPR. Nevertheless, our exposure ascertainment method can also represent a limitation. Biopsy is not always the primary method for diagnosing *H. pylori* infection, as non-invasive procedures (e.g., urea breath test, serology) are also commonly used, especially in young individuals, and some patients may even be diagnosed on a clinical basis without prior testing. *H. pylori* infection is often asymptomatic and, although less likely, this may also apply to gastritis and peptic ulcer. Thus, biopsy-confirmed definition of *H. pylori*, peptic ulcer, and gastritis might have led to under-ascertainment and over-representation of more severe cases. Another factor potentially influencing our results is that individuals experiencing early manifestations of PD may have undergone endoscopy that accidentally detected asymptomatic *H. pylori* infection, increasing the magnitude of our risk estimates. However, we tried to account for this by adjusting for number of healthcare contacts before the index date, thus we believe that any misclassification in this study is non-differential and would potentially bias our estimates toward the null. In case of differential misclassification, we would expect higher risk estimates for PD closer to the diagnosis, but we did not observe any time-varying effects for *H. pylori.* The use of ICD codes for outcome as certainment is another study limitation. While the accuracy of PD diagnoses in the register is high, the risk of misclassification cannot be ruled out.^36^ The use of COPD as a proxy for smoking status is also a limitation, as it relies on ICD codes and mostly captures heavy smokers. Lastly, despite the matched design and the effort to adjust for relevant covariates, residual confounding might have contributed to our findings, as we lacked data on potentially relevant confounders such as lifestyle factors.

In conclusion, our findings suggest that *H. pylori infection* and gastritis may have some relevance for PD but are unlikely to be major contributors to its pathogenesis. Future studies are needed to clarify whether the associations we found reflect a true causal relationship, prodromal PD symptoms, or shared underlying mechanisms influencing both gastrointestinal impairment and neurodegeneration. Considering other factors related to the gut-brain axis, such as microbiome composition or lifestyle habits, might help to investigate the complex interplay underlying PD pathogenesis further.

## Supplemental Material

sj-docx-1-pkn-10.1177_1877718X261466875 - Supplemental material for *Helicobacter pylori* infection, peptic ulcer, gastritis and risk of Parkinson's disease: A nationwide matched cohort study Supplemental material, sj-docx-1-pkn-10.1177_1877718X261466875 for *Helicobacter pylori* infection, peptic ulcer, gastritis and risk of Parkinson's disease: A nationwide matched cohort study by Sara Licaj, Alexander Ploner, Agnieszka Szwajda, Sara Hägg, Fang Fang, Jonas F Ludvigsson and Karin Wirdefeldt in Journal of Parkinson's Disease
