# Supplementary material for: Helicobacter pylori infection, peptic ulcer, gastritis and risk of Parkinson's disease: A nationwide matched cohort study
Source: J Parkinsons Dis. 2026 Jul 15;16(6):1164–73. doi: 10.1177/1877718X261466875 (PMC13443069; doi:10.1177/1877718X261466875)
Supplement: Supplementary file 1 [file sj-docx-1-pkn-10.1177_1877718X261466875.docx]

**Supplementary material**

**Table S1**. Exposure patterns in the analysed ESPRESSO cohorts

**Table S2**. Full results of the multivariate Cox regression analysis stratified by matched sets with attained age as the primary time scale

**Table S3**. Comparison between *H. pylori*, gastritis and peptic ulcer diagnoses in ESPRESSO and the National Patient Register (NPR)

**Table S4**. Results of the sensitivity analysis: multivariate Cox regression stratified by matched sets with attained age as the primary time scale and primary PD diagnoses as the main outcome

| **Table S1.** Exposure patterns in the analysed ESPRESSO cohorts. \| \| \| \|; ***H. pylori*** \| \| \| \|; Valid \| n \| Invalid \| n \|; H.pyl-_H.pyl-_H.pyl-_H.pyl-_H.pyl+ \| 1 \| H.pyl-_H.pyl+_H.pyl- \| 11 \|; H.pyl-_H.pyl-_H.pyl-_H.pyl+ \| 1 \| H.pyl+_H.pyl- \| 258 \|; H.pyl-_H.pyl-_H.pyl-_H.pyl- \| 14 \| \| \|; H.pyl-_H.pyl-_H.pyl+ \| 67 \| \| \|; H.pyl-_H.pyl-_H.pyl- \| 367 \| \| \|; H.pyl-_H.pyl- \| 9,829 \| \| \|; H.pyl-_H.pyl+ \| 2,063 \| \| \|; **Peptic ulcer** \| \| \| \|; Valid \| n \| Invalid \| n \|; Pept.Ulcer-_Pept.Ulcer-_Pept.Ulcer-_Pept.Ulcer-_Pept.Ulcer- \| 1 \| Pept.Ulcer-_Pept.Ulcer+_Pept.Ulcer- \| 8 \|; Pept.Ulcer-_Pept.Ulcer-_Pept.Ulcer-_Pept.Ulcer- \| 12 \| Pept.Ulcer+_Pept.Ulcer- \| 172 \|; Pept.Ulcer-_Pept.Ulcer-_Pept.Ulcer+ \| 53 \| \| \|; Pept.Ulcer-_Pept.Ulcer-_Pept.Ulcer- \| 332 \| \| \|; Pept.Ulcer-_Pept.Ulcer+ \| 1,967 \| \| \|; Pept.Ulcer-_Pept.Ulcer- \| 10,234 \| \| \|; **Gastritis** \| \| \| \|; Valid \| n \| Invalid \| n \|; Gastritis-_Gastritis-_Gastritis-_Gastritis-_Gastritis-_Gastritis+ \| 2 \| Gastritis-_Gastritis-_Gastritis+_Gastritis-_Gastritis- \| 1 \|; Gastritis-_Gastritis-_Gastritis-_Gastritis-_Gastritis-_Gastritis- \| 3 \| Gastritis+_Gastritis-_Gastritis-_Gastritis- \| 1 \|; Gastritis-_Gastritis-_Gastritis-_Gastritis-_Gastritis+ \| 20 \| Gastritis-_Gastritis-_Gastritis-_Gastritis+_Gastritis- \| 1 \|; Gastritis-_Gastritis-_Gastritis-_Gastritis-_Gastritis- \| 92 \| Gastritis-_Gastritis+_Gastritis-_Gastritis- \| 6 \|; Gastritis-_Gastritis-_Gastritis-_Gastritis+ \| 275 \| Gastritis+_Gastritis-_Gastritis- \| 39 \|; Gastritis-_Gastritis-_Gastritis-_Gastritis- \| 1,178 \| Gastritis-_Gastritis-_Gastritis+_Gastritis- \| 45 \|; Gastritis-_Gastritis-_Gastritis+ \| 3,192 \| Gastritis-_Gastritis+_Gastritis- \| 446 \|; Gastritis-_Gastritis-_Gastritis- \| 13,076 \| Gastritis+_Gastritis- \| 3,194 \|; Gastritis-_Gastritis+ \| 28,631 \| \| \|; Gastritis-_Gastritis- \| 125,416 \| \| \|; Individuals who entered the study as unexposed could be matched to multiple exposed individuals during follow-up. To avoid counting their time at risk more than once in the statistical analysis, we censored reused referents one day before they were re-indexed, e.g., when they were assigned a new index date in another matched set. Individuals who entered the study as unexposed, but became exposed while still at risk for PD, contributed both unexposed risk time (from inclusion to the day before exposure diagnosis) and exposed risk time (from the exposure date onwards), treating exposure as time-varying covariate. Referents who had previously served as index persons were excluded from re-indexing, though we included their exposed risk time as index person in the analysis. \| \| \| \| **Table S2.** Full results of the multivariate Cox regression analysis stratified by matched sets with attained age as the primary time scale. \| \| \| \| \| \| \| \| \| \| \|; ***H. pylori*** \| **HR**^1^ \| **95% CI**^1^ \| **p-value** \| **Peptic ulcer** \| **HR**^1^ \| **95% CI**^1^ \| **p-value** \| **Gastritis** \| **HR**^1^ \| **95% CI**^1^ \| **p-value**; **Status** \| \| \| \| \| \| \| \| \| \| \|; *H. pylori-* \| — \| — \| \| Peptic ulcer- \| — \| — \| \| Gastritis- \| — \| — \|; *H. pylori+* \| 1.14 \| 1.02, 1.28 \| 0.026 \| Peptic ulcer+ \| 1.08 \| 0.98, 1.19 \| 0.12 \| Gastritis+ \| 1.14 \| 1.09, 1.19 \| <0.001; **Education** \| \| \| \| \| \| \| \| \| \| \|; ≤ 9 years \| — \| — \| \| \| — \| — \| \| \| — \| — \|; 10–12 years \| 0.98 \| 0.87, 1.09 \| 0.7 \| \| 1.09 \| 1.00, 1.19 \| 0.060 \| \| 1.08 \| 1.03, 1.13 \| 0.001; ≥ 13 years \| 1.16 \| 1.02, 1.33 \| 0.026 \| \| 1.08 \| 0.97, 1.21 \| 0.2 \| \| 1.24 \| 1.17, 1.31 \| <0.001; Missing \| 0.77 \| 0.50, 1.17 \| 0.2 \| \| 1.24 \| 0.94, 1.65 \| 0.13 \| \| 1.06 \| 0.90, 1.24 \| 0.5; **Country of Birth** \| \| \| \| \| \| \| \| \| \| \|; Sweden \| — \| — \| \| \| — \| — \| \| \| — \| — \|; Other \| 0.91 \| 0.78, 1.05 \| 0.2 \| \| 0.89 \| 0.78, 1.02 \| 0.10 \| \| 0.90 \| 0.84, 0.96 \| 0.002; **COPD**² \| 0.78 \| 0.61, 1.00 \| 0.051 \| \| 0.75 \| 0.61, 0.91 \| 0.003 \| \| 0.78 \| 0.71, 0.87 \| <0.001; **N. of healthcare contacts**³ \| \| \| \| \| \| \| \| \| \| \|; 0 \| — \| — \| \| \| — \| — \| \| \| — \| — \|; 1 \| 0.80 \| 0.69, 0.92 \| 0.002 \| \| 1.16 \| 1.05, 1.29 \| 0.003 \| \| 1.10 \| 1.04, 1.17 \| <0.001; 2 \| 1.00 \| 0.84, 1.18 \| >0.9 \| \| 1.24 \| 1.09, 1.41 \| <0.001 \| \| 1.16 \| 1.08, 1.25 \| <0.001; 3-5 \| 0.90 \| 0.77, 1.05 \| 0.2 \| \| 1.26 \| 1.12, 1.42 \| <0.001 \| \| 1.19 \| 1.12, 1.27 \| <0.001; ≥ 6 \| 1.19 \| 1.02, 1.38 \| 0.030 \| \| 1.38 \| 1.20, 1.59 \| <0.001 \| \| 1.38 \| 1.28, 1.48 \| <0.001; ^1^HR: Hazard Ratio, CI: Confidence Interval ²Number of chronic obstructive pulmonary disease (COPD) diagnoses at baseline or during follow-up. ³Number of inpatient and outpatient healthcare contacts, within 5 years and up to 6 months before the index date. \| \| \| \| \| \| \| \| \| \| \| **Table S3.** Comparison between *H. pylori*, gastritis and peptic ulcer diagnoses in ESPRESSO and the National Patient Register (NPR)^1^ \| \| \|; \| ***Has an NPR diagnosis^2^*** ***n (%)*** \| \|; \| *No* \| *Before index date in ESPRESSO* \| *After index date in ESPRESSO*; ***ESPRESSO status*** \| \| \|; *H. pylori-* \| 94,935 (99.9) \| 8 (0.008) \| 79 (0.1); *H. pylori+* \| 17,578 (96.3) \| 276 (1.5) \| 404 (2.2); Gastritis*-* \| 1,295,205 (95.4) \| 13,048 (1.0) \| 48,646 (3.6); Gastritis+ \| 206,430 (75.9) \| 32,472 (11.9) \| 33,064 (12.2); Peptic ulcer*-* \| 277,415 (94.7) \| 3,475 (1.2) \| 12,140 (4.1); Peptic ulcer*+* \| 18,865 (34.8) \| 15,212 (28.0) \| 20,201 (37.2); ^1^ We compared the first diagnosis in the NPR with the first diagnosis in ESPRESSO. ^2^ We retrieved *H. pylori* infection diagnoses in the NPR with the ICD-10 code “B980”, in combination with any other code starting with “K2”. The first NPR diagnosis was in 2010, thus we only considered ESPRESSO diagnoses after January 1^st^ 2010. For peptic ulcer, we identified NPR diagnoses with the ICD-10 codes “K25” (gastric ulcer), “K26” (duodenal ulcer) and “K27” (peptic ulcer) and “531”, “532” and “533” ICD-9 codes. For gastritis, we retrieved NPR diagnoses with the ICD-10 code “K29” and the ICD-9 code “535”. \| \| \| | | | | | | | | | | | |
| --- | --- | --- | --- | --- | --- | --- | --- | --- | --- | --- | --- |
| **Table S4.** Results of the sensitivity analysis: multivariate Cox regression stratified by matched sets with attained age as the primary time scale and primary PD diagnoses as the main outcome. The number of primary PD diagnoses was 1,604 in the *H. pylori* cohort, 2,556 in the peptic ulcer cohort, and 9,390 in the gastritis cohort. | | | | | | | | | | | |
| ***H. pylori*** |  |  |  | **Peptic ulcer** |  |  |  | **Gastritis** |  |  |  |
|  | **HR**^1^ | **95% CI**^1^ | **p-value** |  | **HR**^1^ | **95% CI**^1^ | **p-value** |  | **HR**^1^ | **95% CI**^1^ | **p-value** |
| **Status** |  |  |  |  |  |  |  |  |  |  |  |
| *H. pylori-* | — | — |  | Peptic ulcer- | — | — |  | Gastritis- | — | — |  |
| *H. pylori+* | 1.06 | 0.92, 1.22 | 0.5 | Peptic ulcer+ | 1.03 | 0.90, 1.17 | 0.7 | Gastritis+ | 1.13 | 1.07, 1.20 | <0.001 |
| **Education** |  |  |  |  |  |  |  |  |  |  |  |
| ≤ 9 years | — | — |  |  | — | — |  |  | — | — |  |
| 10–12 years | 0.96 | 0.84, 1.10 | 0.6 |  | 1.15 | 1.03, 1.28 | 0.013 |  | 1.15 | 1.08, 1.21 | <0.001 |
| ≥ 13 years | 1.24 | 1.06, 1.44 | 0.006 |  | 1.22 | 1.06, 1.40 | 0.005 |  | 1.38 | 1.29, 1.48 | <0.001 |
| Missing | 0.50 | 0.24, 1.01 | 0.052 |  | 0.81 | 0.51, 1.27 | 0.4 |  | 0.90 | 0.70, 1.15 | 0.4 |
| **Country of Birth** |  |  |  |  |  |  |  |  |  |  |  |
| Sweden | — | — |  |  | — | — |  |  | — | — |  |
| Other | 0.83 | 0.70, 0.98 | 0.033 |  | 0.92 | 0.78, 1.09 | 0.3 |  | 0.91 | 0.84, 0.99 | 0.021 |
| **COPD**² | 0.58 | 0.42, 0.82 | 0.002 |  | 0.58 | 0.44, 0.75 | <0.001 |  | 0.61 | 0.53, 0.71 | <0.001 |
| **N. of healthcare contacts**³ | | |  |  |  |  |  |  |  |  |  |
| 0 | — | — |  |  | — | — |  |  | — | — |  |
| 1 | 0.79 | 0.67, 0.94 | 0.008 |  | 1.09 | 0.95, 1.23 | 0.2 |  | 1.02 | 0.95, 1.09 | 0.6 |
| 2 | 0.96 | 0.78, 1.18 | 0.7 |  | 1.09 | 0.92, 1.29 | 0.3 |  | 1.06 | 0.97, 1.16 | 0.2 |
| 3-5 | 0.92 | 0.76, 1.11 | 0.4 |  | 1.22 | 1.04, 1.43 | 0.015 |  | 1.02 | 0.94, 1.11 | 0.6 |
| ≥ 6 | 1.03 | 0.85, 1.24 | 0.8 |  | 1.18 | 0.99, 1.42 | 0.072 |  | 1.08 | 0.99, 1.18 | 0.10 |
| ^1^HR: Hazard Ratio, CI: Confidence Interval  ²Number of chronic obstructive pulmonary disease (COPD) diagnoses at baseline or during follow-up.  ³Number of inpatient and outpatient healthcare contacts, within 5 years and up to 6 months before the index date. | | | | | | | | | | |  |
